# AR facilitates YAP-TEAD interaction with the AM promoter to enhance mast cell infiltration into cutaneous neurofibroma

**DOI:** 10.1038/s41598-019-56022-9

**Published:** 2019-12-18

**Authors:** Jing Jia, Haibao Zhang, Hongke Zhang, Wenbo Liu, Huicong Du, Maoguo Shu, Lin He

**Affiliations:** 1 Department of Plastic, Cosmetic and Maxillofacial Surgery, The First Affiliated Hospital of Xi’an Jiaotong University, Xi’an, Shaanxi China; 2 The School of Electronic and Information Engineering, Xi’an Jiaotong University, Xi’an, Shaanxi China; 3 Key Laboratory for Tumor Precision Medicine of Shaanxi Province, Xi’an, Shaanxi China

**Keywords:** Mechanisms of disease, Skin diseases

## Abstract

Abundant mast cell infiltration and disease initiation at puberty are hallmark features of cutaneous neurofibroma (cNF). However, the association between mast cell infiltration and steroid hormones in cNF remains unclear. Here, we determined that androgen receptor (AR) expression is positively associated with mast cell density in cNF tissues. Moreover, both *in vitro* cell experiments and *in vivo* mouse models verified that activated AR promoted mast cell infiltration and that AR inhibition reduced mast cell infiltration. Analyses in cell models and xenograft tumours both demonstrated that AR upregulated Yes associate  protein 1 (YAP)-adrenomedullin (AM) signalling. Clinical samples from cNF patients further verified that AR was positively related to YAP and AM. Mechanistic analysis revealed that AR accelerates AM transcription *via* enhancing YAP- TEA domain transcription factor (TEAD) binding to the AM promoter. Consequently, the upregulated AM enhanced mast cell recruitment. Interruption of the YAP-TEAD interaction or inhibition of AM could impair mast cell accumulation induced by active AR, which indicated that this newly found signalling pathway may provide novel targets for cNF treatment.

## Introduction

Neurofibromatosis type 1 (NF1) is induce by Nf1 mutation with a morbidity rate of 1:2600–1:4500 worldwide^1^. NF1 has nearly complete penetrance with various spectrum of manifestations. Neurofibroma is the main type of NF1, including cutaneous neurofibromas (cNFs), plexiform neurofibromas (pNFs) and malignant peripheral nerve sheath tumours (MPNSTs). Although cNFs rarely becomes malignant, they appear in almost all NF1 patients with vast differences in number and size^2^. These cNFs cause itching, pain and cosmetic burdens that are linked to psychosocial changes^3^. Unfortunately, treatments for cNF are limited to surgery and laser excision, which is challenging with numerous or large neoplasias.

Unlike other types of neurofibromas, cNF is usually remains dormant at birth, appears at puberty and worsens with age. Some patients showed increased progression of cNF and upregulated malignant potential of pNF during pregnancy^4,5^. These data suggest that steroid hormones may play a role in neurofibroma initiation, development and maintenance. Oestrogen receptor and progesterone receptor have been detected in rat Schwann cells^6^, while the expression and function of the androgen receptor (AR) in neurofibroma cells require further elucidation. The quiescent stage of cNF, before puberty for example, may provide a chance to limit cNF progression. Thus, discovering the role of steroid hormones in cNF may provide clues regarding ways to limit cNF development.

The cells that compose cNF include Schwann cells, endothelial cells, fibroblasts and inflammatory cells^7^. Anecdotal evidence has shown that injury-associated inflammation facilitates neurofibroma formation in mouse models^8^ and NF1 patients^9^. Moreover, loss of Nf1 gene could enhance inflammatory gene expression in cultured Schwann cells^10^. As a major component of the inflammatory response, infiltrated mast cells secrete proteins to remodel extracellular matix^11^ and growth factors to promote tumour progression^12^. Consistently, the removal of mast cells from the microenvironment impaired tumour initiation and induced shrinkage of neurofibromas^13,14^. Therefore, targeting of mast cells *via* KIT was used for neurofibroma clinical treatment and achieved some success^15^. However, some patients failed to respond to KIT inhibition^15^. It thus emerges that there are additional elements mediating mast cell accumulation.

Here, we found that active AR facilitated mast cell infiltration *via* accelerating the interaction of the YAP-TEAD complex with the adrenomedullin (AM) promoter. As both steroid hormones and YAP play important roles in mediating mast cell activity, the therapeutic potency of targeting the newly investigated pathway to suppress mast cell infiltration is worth further exploration.

## Results

### Mast cell infiltration was strongly associated with AR expression in cNF tissue

**Figure 1:**
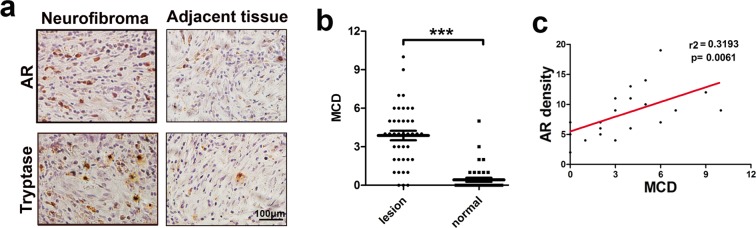
Enhanced mast cell infiltration positively correlated with upregulated AR expression in cNF tissues. Forty cNFs and adjacent soft tissue samples were immunohistochemically stained for tryptase and AR. Each section was examined under a high-power field (×400) in a double-blinded manner. Mast cell density (MCD) was calculated as the average measurement of 10 random fields. (**a**) Representative photograph of tryptase-positive mast cells and AR in cNF and adjacent normal dermal tissues. (**b**) MCD in neurofibroma and adjacent soft tissue. (**c**) Correlation analysis of AR expression and MCD by linear regression. ***p < 0.001.

To investigate the potential association of AR expression and mast cell infiltration, the major immune cells in the cNF tumour microenvironment (TME) were subjected to immunohistochemistry (IHC) analyses with anti-tryptase (specific marker of mast cells)^16^ and anti-AR antibodies in 40 cNF tissues and adjacent normal tissues. The results revealed that mast cell density (MCD) was significantly increased in cNF tissues compared to adjacent normal tissues (3.875 ± 0.369 per high power field (HPF) vs 0.425 ± 0.1597 per HPF, P < 0.001, Fig. 1a,b). AR staining indicated overexpression of AR in cNF tissue (Fig. 1a). In addition, analysis of cNF tissue from 22 male patients suggested that MCD increased with AR expression in cNF tissue (Fig. 1c). Furthermore, no difference was found in MCD in 22 male and 18 female NF1 patients (3.727 ± 0.578 per HPF vs 3.944 ± 0.4463 per HPF, P = 0.7756, Supplementary Fig. S1b), which indicated that sex does not impact mast cell infiltration. Linear regression analysis showed no relationship between MCD and NF1 patient age (r = 0.147, P = 0.36, Supplementary Fig. S1c).

Taken together, the results from Fig. 1a–c indicated that infiltrating mast cells were significantly increased and positively correlated with robust AR expression in cNF tissues from male patients.

### Activated AR promoted mast cell recruitment *in vivo* and *in vitro*

**Figure 2:**
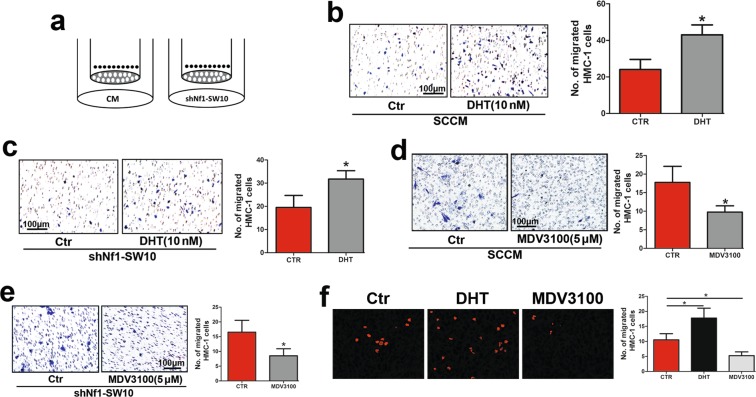
AR activation promoted mast cell infiltration. (**a**) Conditioned medium from 2 × 10^6^ shNf1 -SW10 cells (SCCM) or 2 × 10^6^ shNf1 -SW10 cells was added to the lower chamber of a Transwell system. HMC-1 cells were added to the upper chamber to evaluate the recruitment of mast cells. Cartoon for HMC-1 recruitment. (**b**) Enhanced HMC-1 migration was found when conditioned medium from DHT (dihydrotestosterone, 10 μM)-treated SW10 cells were plated in the lower chamber; Right panel: quantification of migrated HMC-1 cells. (**c**) Augmented HMC-1 cell infiltration was found when DHT-treated shNf1-SW10 cells were plated in the lower chamber compared with when control cells were plated in the lower chamber. Right: quantification of HMC-1 cell migration. (**d**) Conditioned medium from MDV3100-treated SW10 cells attracted fewer HMC-1 cells than the control group; Right panel: quantification of HMC-1 cells. (**e**) MDV3100-treated SW10 cells showed impaired recruitment of HMC-1 cells; Right panel: quantification of recruited HMC-1 cells. (**f**) RFP-labelled HMC-1 cells were constituted and injected *via* the caudal veins of mice receiving different treatments. HMC-1 cells in the frozen sections of the tumours were detected and analysed with fluorescence microscopy; Right panel: quantification of RFP-labelled HMC-1 cells in tumours. *P < 0.05.

The Nf1 mutation, which causes a nonfunctional copy of the Nf1 gene, is the chief genetic change in neurofibromatosis type 1^13^. Therefore, we constructed a cell model of cNF by knocking down Nf1 in SW10 cells in this study (Supplementary Fig. S1a). To determine the potential association of upregulated AR expression and increased mast cell infiltration in cNF tissues, we performed a mast cell recruitment assay with SW10 cells or conditioned medium from SW10 cells (SCCM) in the lower chamber (Fig. 2a). As shown in Fig. 2b,c, exaggerated HMC-1 migration was found in DHT-treated SW10 cells or medium from DHT-treated SW10 cells in the lower chamber. Next, we employed MDV3100, an inhibitor of AR also called enzalutamide, to treat SW10 cells. Consistently, both MDV3100-treated SW10 cells and medium from these cells showed a tempered ability to recruit HMC-1 cells (Fig. 2d,e).

To further determine whether activated AR contributed to mast cell infiltration into neurofibroma, 2 × 10^6^ shNf1-SW10 cells were subcutaneously injected into the right dorsal region of male nude mice. Mice were randomly divided into three groups and treated with DHT (0.6 mg/kg), MDV3100 (1 mg/kg) or DMSO by intraperitoneal injection. Red fluorescent protein (RFP)-labelled HMC-1 cells were constituted and injected through the caudal vein. Forty-eight hours later, tumours were harvested, and RFP-labelled HMC-1 was observed to be increased in DHT-treated tumours and decreased in MDV3100-treated tumours compared to DMSO-treated tumours (Fig. 2f).

Taken together, SW10 cells with activated AR could increase mast cell infiltration into the tumour *in vivo* and *in vitro*.

### Activated AR upregulated YAP and AM to promote mast cell recruitment

**Figure 3:**
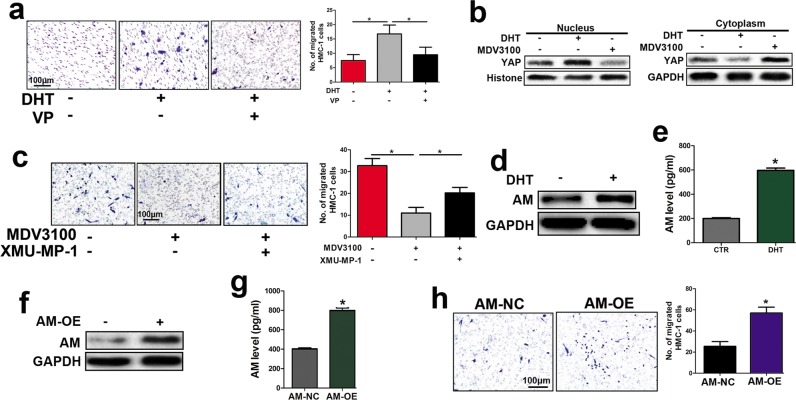
AR activated YAP and upregulated AM to recruit HMC-1 cells. (**a**) DHT-treated SW10 cells induced enhanced HMC-1 cell migration, and VP (5 μM) impaired the enhancement. Right panel: quantification of migrated HMC-1 cells. (**b**) DHT increased the nuclear YAP expression, and MDV3100 decreased the nuclear YAP expression in SW10 cells. (**c**) MDV3100 weakened HMC-1 infiltration, and XMU-MP-1 (5 μM) impaired this weakening. Right panel: quantification of migrated HMC-1 cells. (**d**,**e**) SW10 cells treated with DHT presented robust AM expression by Western blotting and ELISA. (**f**) We overexpressed AM with lentivirus and analysed the protein level of AM in SW10 cells. (**g**) Concentration of secreted AM in the medium of AM-overexpressing (AM-OE) and negative control (AM-NC) SW10 cells. (**h**) AM-OE SW10 cells recruited more HMC-1 cells than did AM-NC SW10 cells; Right panel: quantification of recruited HMC-1 cells. *P < 0.05.

Next, we explored the potential pathways that mediate AR-driven HMC-1 recruitment. Except for YAP, inhibition of PI3K/AKT pathway, transforming growth factor-β (TGF-β), JAK/Stat3 pathway and MAPK pathway components all failed to reverse DHT-induced HMC-1 recruitment (Supplementary Fig. S2 and Fig. 3a).Verteporfin (VP) is toxic to cells, which may decrease the cell number of shNf1-SW10 cells to impair mast cell recruitment. Thus, we used the CCK8 assay and detected slightly decreased cell viability in VP-treated shNf1-SW10 cells compared to control cells (Supplementary Fig. S3). Considering the significantly decreased HMC-1 recruitment induced by VP treatment of shNf1-SW10 cells and the important role of YAP-driven signalling in schwannoma proliferation^17^ and cNF tumorigenesis^18^, we focused on the role of YAP in DHT-induced HMC-1 recruitment. We observed robust nuclear YAP expression in DHT-treated SW10 cells and weakened nuclear YAP in MDV3100-treated SW10 cells, which demonstrated that AR regulates nuclear YAP. YAP in nucleus might further modulate target protein expression (Fig. 3b). Furthermore, accumulation of YAP was detected in SW10 cells treated with MDV3100, while XMU-MP-1 treatment, which inhibits MST1/2 to enhance YAP efficacy, impaired the diminishment (Fig. 3c).

To further investigate cytokines and/or chemokines associated with mast cell recruitment, we performed a qPCR assay and found that AM was significantly upregulated in DHT-treated SW10 cells compared to control cells (Supplementary Fig. S4). Similar results were obtained with ELISA and Western blot assays, which showed augmented AM levels in DHT-treated SW10 cells (Fig. 3d,e). To identify the role of AM in SW10-induced mast cell recruitment, we overexpressed AM (AM-OE) in SW10 cells (Fig. 3f), which led to increased secretion of AM into the medium of SW10 cells compared with that in the control cells (Fig. 3g). As a result, AM overexpression in SW10 cells enhanced mast cell recruitment (Fig. 3h).

Taken together, DHT treatment promoted YAP activity and AM expression to facilitate mast cell recruitment. Moreover, AM played an important role in SW10-induced mast cell recruitment.

### Activated AR recruited mast cells via YAP-AM signalling

**Figure 4:**
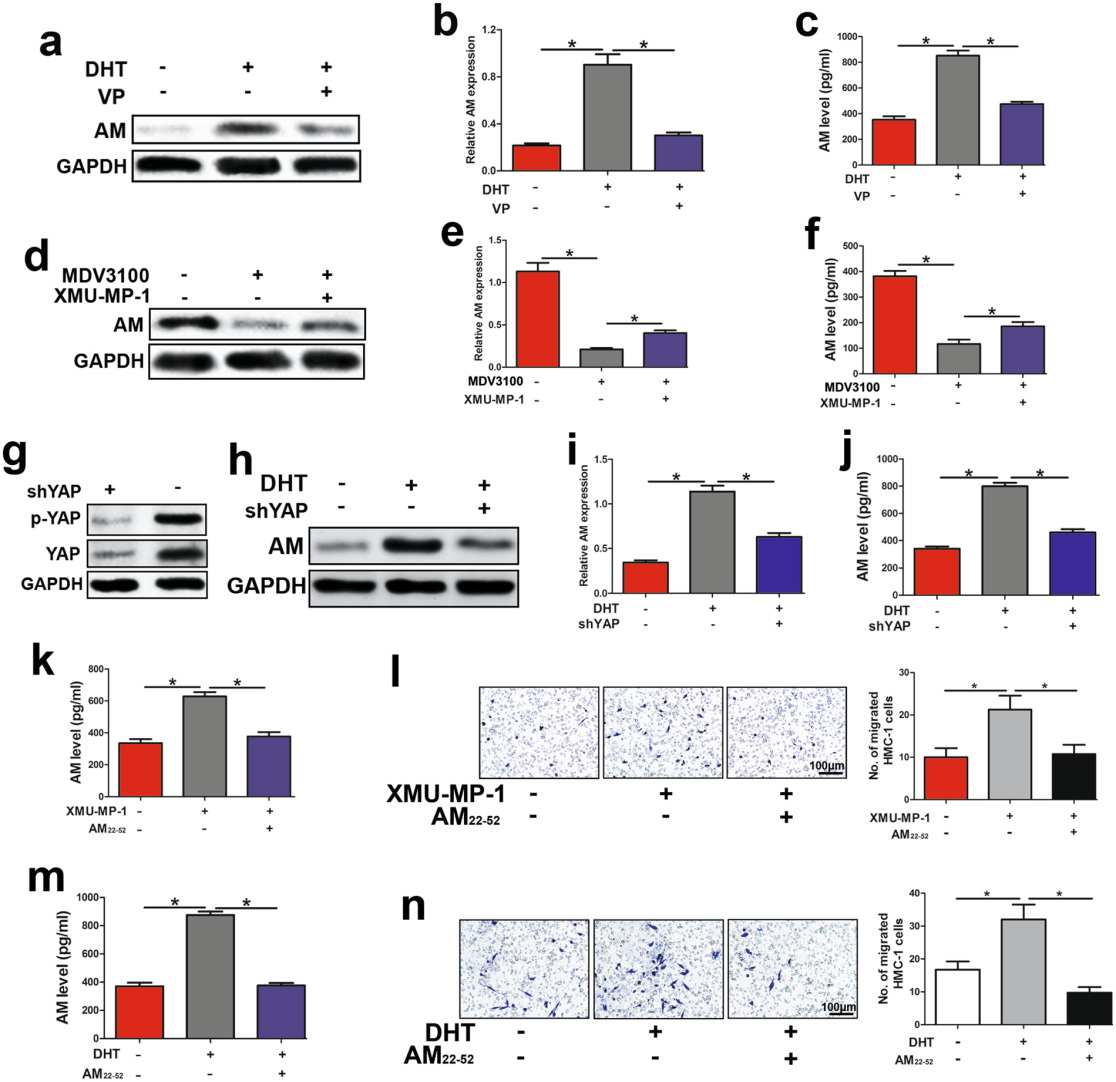
AR facilitated mast cell accumulation *via* YAP-AM signalling. (**a**,**b**) DHT upregulated AM at the protein level and mRNA level, while VP (5 μM) repressed the upregulation. (**c**) Accelerated AM secretion was found in DHT-stimulating SW10 cells, and VP tempered the acceleration. (**d** and **e**) MDV3100 decreased AM protein levels and mRNA levels, while XMU-MP-1 reversed this decrease. (**f**) MDV3100 repressed AM concentration in medium of SW10 cells, and XMU-MP-1 impaired the repression. (**g**) Lentivirus carrying shRNA targeting YAP was used to knockdown YAP in shNf1-SW10 cells, and the protein levels of YAP and p-YAP were detected. (**h-j**) Western blot assay, qPCR assay and ELISA detected that DHT treatment upregulated AM in shNf1-SW10 cells and that YAP knockdown reduced the upregulation. (**k**) XMU-MP-1 accelerated AM expression. (**l**) Enhanced HMC-1 accumulation was found in XMU-MP-1-treated SW10 cells, and AM_22–52_ suppressed the enhancement; Right panel: quantification of migrated HMC-1 cells. (**m**) AM_22–52_ attenuated the increase in secreted AM induced by DHT treatment. (**n**) AM_22–52_ weakened DHT-induced HMC-1 infiltration; Right panel: quantification of migrated HMC-1 cells. *P < 0.05.

To determine whether AR upregulated AM through activating YAP, we treated SW10 cells with VP (5 μM) and DHT. The results of Western blot assays and qPCR assays validated that VP reduced DHT-induced AM expression and AM concentration in the media (Fig. 4a–c). By applying the opposite approach, we also dissected the reduction of AM in SW10 cells treated with MDV3100; simultaneous treatment with XMU-MP-1 impaired that reduction (Fig. 4d–f). For further confirmation, we knocked down YAP in shNf1-SW10 cells (Fig. 4g). Western blot assays, qPCR assays and ELISA showed that YAP knockdown reduced DHT-induced AM upregulation in shNf1-SW10 cells (Fig. 4h–j). These results reveal that AR positively regulated AM by modulating YAP.

Furthermore, we treated SW10 cells with XMU-MP-1, and these cells secreted higher levels of AM and recruited more HMC-1 cells. By treating cells with AM_22–52_, a specific antagonist of AM, we detected reduced XMU-MP-1-induced AM secretion and HMC-1 recruitment (Fig. 4k,l). Consistently, AM_22–52_ also attenuated DHT-induced AM secretion and HMC-1 accumulation (Fig. 4m,n).

Taken together, the results of Fig. 4a–n indicate that YAP was important in AR-mediated AM expression and sequential HMC-1 cell recruitment.

### AR activation upregulated AM transcription via enhancing YAP and TEAD binding to the promoter of AM

**Figure 5:**
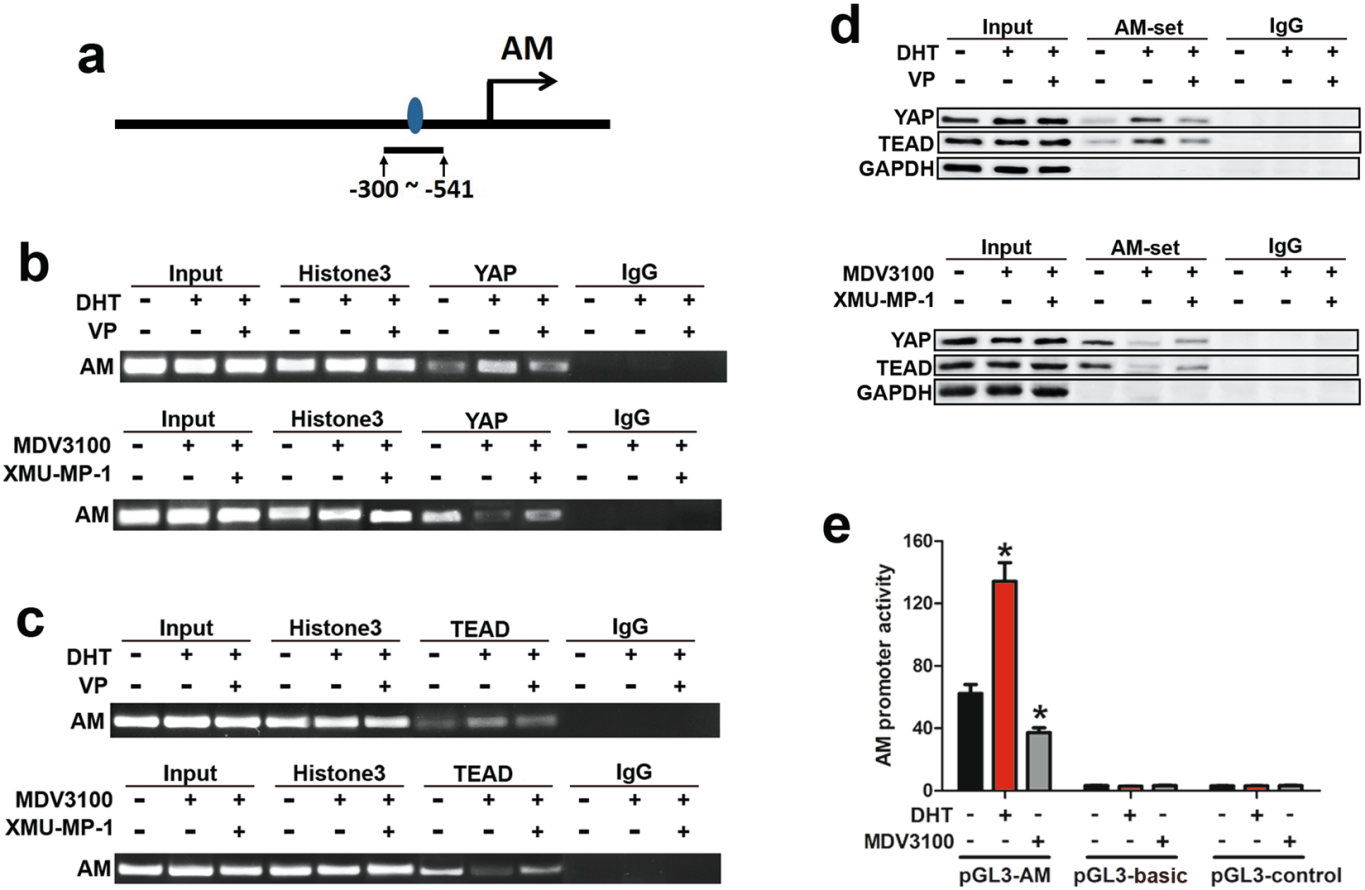
AR promoted YAP-TEAD binding to the promoter of AM and facilitated its transcription. (**a**) Cartoon of the YAP-TEAD binding region in the AM promoter for mapping oligonucleotide pulldown assays and ChIP assays. (**b**,**c**) Both YAP and TEAD bound to the AM promoter in SW10 cells. DHT (10 μM) enhanced the binding, and MDV3100 (5 μM) diminished the binding, as detected by ChIP assay. (**d**) Binding of YAP and TEAD on the AM promoter, as detected by oligonucleotide pull down assays and Western blot analysis. (**e**) DHT enhanced the activity of the AM promoter driven by YAP, and MDV3100 suppressed promoter activity. *P < 0.05.

Our results in Fig. 4 show that YAP regulates AM at the mRNA level. In addition, previous studies have shown that YAP accumulates in the nucleus where it forms a complex with TEAD to activate a panel of targeted genes^19^. Thus, we hypothesized that YAP cooperated with a TEAD family member to regulate AM expression. Therefore, we predicted the potential binding site of TEAD in the AM promoter and found the regions that may possess the highest binding affinity. Hence, AM promoter regions containing the TEAD binding site were selected (Fig. 5a), and primers for that binding region were designed. Chromatin immunoprecipitation (ChIP) assays showed that both YAP and TEAD could bind to the promoter of AM. Moreover, DHT treatment robustly inhibited binding, and MDV3100 treatment diminished binding (Fig. 5b,c).

For further affirmation, the binding region of the AM promoter was identified and amplified with biotinylated primers (Supplementary Table S3). As detected by oligonucleotide pull-down assays and Western blot analysis, the AM promoter could precipitate both YAP and TEAD. Accordingly, the relative amount of precipitated YAP and TEAD was increased with DHT treatment and decreased with MDV3100 treatment (Fig. 5d). To further clarify the hypotheses, we cloned a DNA fragment of the AM promoter and inserted it into a luciferase reporter plasmid for use in dual luciferase activity assays in SW10 cells. Compared to control treatment, DHT treatment enhanced the activity of the AM promoter, while MDV3100 treatment reduced the activity (Fig. 5e).

Taken together, the results from Fig. 5a–e demonstrate that YAP, together with TEAD, elevated AM expression *via* binding to the AM promoter, and this was enhanced by AR activation.

### AR-YAP-AM signalling correlated with mast cell infiltration in clinical cNF samples and xenograft tumour samples

**Figure 6:**
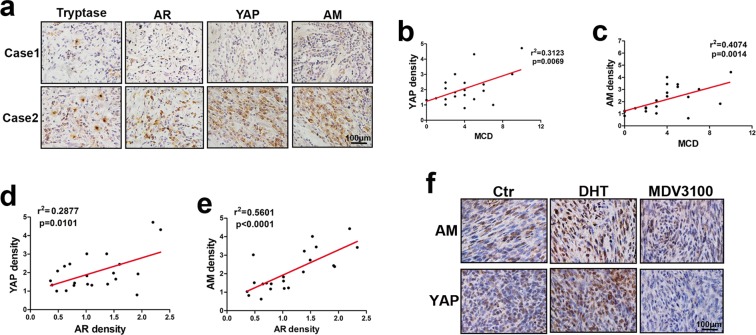
AR promoted YAP-AM-mast cell infiltration *in vivo*. (**a**) Representative images of IHC staining with anti-AR, anti-YAP, anti-AM and anti-tryptase in cNF tissues. (**b**) Correlation analysis of YAP density and mast cell density (MCD). (**c**) Correlation analysis of AM density and MCD. (**d** and **e**) Liner regression analysis of AR density and YAP or AM density. (**f**) Representative images of IHC staining with anti-YAP and anti-AM in xenograft tumours from mice with different treatments.

To confirm that AR activates YAP to upregulate AM in clinical cNF samples, we evaluated the protein levels of YAP and AM in 22 male cNF patients by IHC staining. The results showed that samples containing more mast cells presented higher levels of AM and YAP in general (Fig. 6a). The expression levels of AM and YAP evaluated by IHC staining were estimated by using a scoring system combining percentage and intensity. In a single field, the intensity was identified as 0 for no staining, 1 for slight staining, 2 for moderate staining and 3 for strong staining. The staining percentage was scored as follows: 0 for no staining, 1 for lower than 25%, 2 for 25% to 50%, 3 for 50% to 75% and 4 for more than 75%. The staining score was defined as the product of staining percentage score and intensity score. The positive correlation between AM expression and MCD was obtained using linear regression analysis (Fig. 6b). Similarly, mast cell infiltration also correlated with YAP expression (Fig. 6c). Moreover, AR expression was correlated with AM and YAP expression in clinical cNF samples (Fig. 6d,e). These results demonstrate that mast cell infiltration was accompanied by elevated AM and YAP expression in cNF patient samples. Further investigation by IHC staining in subcutaneous xenograft tumours also confirmed increased AM and YAP in DHT-treated tumours and decreased AM and YAP in MDV3100-treated tumours (Fig. 6d). Integrating the results in Fig. 2f, these results further suggest that activated AR, together with upregulated AM and YAP, facilitated mast cell recruitment.

Taken together, the results of Fig. 6a–f validate that AR-YAP-AM signalling played a critical role in mast cell infiltration in xenograft tumour samples and clinical patient samples.

## Discussion

Neurofibromatosis type 1 (NF1) has been considered one of the most common genetic disorders. Although few complications associated with NF1 are lethal, benign tumours cause severe disfiguring in the majority of NF1 patients. Fighting neurofibroma has been a continuous effort with limited success. Due to the important role of the TME in neurofibroma development, targeting select inflammatory cells in cNF has attracted some attention. As the predominant inflammatory cell, neurofibroma-associated mast cells are frequently activated, showing elevated levels of local histamine content and circulating serum IgE^20^, which is not common in other types of neoplasms. This unique characteristic makes mast cells a focus in neurofibroma research. KIT, the receptor of stem cell factor (SCF), played an important role in mast cell migration^13^ and inflammatory activity^11^. A KIT inhibitor was used in clinical neurofibroma treatment and clearly relieved neurofibroma burden in some NF1 patients, but other patients failed to respond to treatment^15^. It is possible that the mast cell number does not fall below a required threshold level to prevent tumour development. Alternatively, other factors existing in neurofibroma are responsible for mast cell infiltration. In our results, we found that AM played an important role in cNF-associated mast cell infiltration, which indicated that the disruption of AM production may be another option of cNF treatment.

With the clinical observation that quick progression of cNF usually appeared during puberty and pregnancy, which was followed by quiescence, we hypothesized that elevated levels of specific hormones seen at puberty and pregnancy may act as “triggers” for tumorigenesis. The functions of oestrogen receptor and progesterone receptor have been reported; however, the contribution of AR in neurofibroma progression needs further clarification. Our study demonstrated that increased AR expression was positively related to robust mast cell density in cNF tissues. Consistently, activation of AR in SW10 cells led to the accumulation of more mast cells, which might sequentially facilitate cNF development. Hence, AR inhibition might be a therapeutic target for cNF treatment. Since AR is critical for adolescent growth, the exploration of additional targets that participate in AR modulation of mast cell accumulation is necessary.

Previous clinical reports found that mutated Nf2, a Hippo pathway regulator, leads to the development of cNF in neurofibromatosis type 2 patients^21,22^. Moreover, activated YAP promoted Nf1-related tumorigenesis in a mouse model, while altering the Hippo pathway alone failed to produce tumours^18^. This finding indicated a modifier role of the Hippo pathway in neurofibroma development. In addition, a previous study also showed that YAP regulated AR-dependent genes^23^. In our study, we detected upregulated YAP in DHT-treated SW10 cells, which was positively associated with mast cell infiltration. Further detection revealed that YAP accelerated AM transcription through interacting, in cooperation with TEAD, with the AM promoter. Additionally, inhibiting YAP impaired DHT-induced mast cell infiltration in SW10 cells, which indicated that YAP might be a potential therapeutic target for cNF.

Adrenomedullin is a member of the calcitonin gene peptide superfamily^24^, and its activity is mediated through interacting with heterodimeric receptors consisting of G-protein-coupled receptors and receptor activity-modifying proteins^25^. AM-regulated mast cell infiltration has been reported to modulate the tumour microenvironment^26^. In addition, previous studies have demonstrated the role of AM in accumulating mast cells^27^ and facilitating mast cell activity^28^, but the role of AM in cNF remains unclear. In our study, we investigated whether AM, upregulated by AR-YAP signalling, accumulated mast cells in the microenvironment of cNF. Robust AM expression was also found in xenograft tumours with DHT treatment and cNF clinical samples with greater mast cell infiltration. We further demonstrated that the AM inhibitor reversed AR-induced mast cell infiltration. Our results showing the role of AM in the accumulation of mast cells in cNF may help us to develop novel therapeutics for cNF.

In summary, our studies indicate a positive association between AR expression and mast cell accumulation in cNF tissues. AR activated YAP, upregulated AM secretion and expression, and subsequently promoted mast cell infiltration. Mechanistically, upregulated YAP, together with TEAD, interacted with the AM promoter to facilitate AM expression.

## Methods

### Statement of methods

The relevant guidelines and regulations were followed for all methods used in this study. Approval was obtained from the institutional review board of the First Affiliated Hospital of Xi’an Jiaotong University for all experimental protocols used in this study.

### Reagents and chemicals

The primary antibody against tryptase was obtained from DAKO A/S (Glostrup, Denmark). Primary antibodies against YAP (#14074), androgen receptor (AR) (#5153), pan-TEAD (#13295) and histone (#7631) were purchased from Cell Signaling Technology (Beverly, MA, USA). DHT (dihydrotestosterone) and MDV3100 (enzalutamide) were purchased from Sigma-Aldrich (St. Louis, MO, USA). The primary anti-AM antibody was purchased from Santa Cruz Biotechnology (Paso Robles, CA, USA). All the reagents were reconstituted and stored strictly according to the manufacturers’ protocol.

### Cell culture and stable RNA interference

SW10 cells (the murine Schwann cell line) were cultured in Dulbecco’s Modified Eagle’s Medium (DMEM) with 10% foetal bovine serum (FBS). The HMC-1 cells (human mast cells) were cultured with Iscove’s Modified Dulbecco’s Medium (IMDM) with 10% FBS added.

Cells were maintained in a fully humidified atmosphere (5% CO_2_, 37 °C). Replication-defective lentiviruses containing short hairpin RNA (shRNA) targeting specific gene or nonsilencing control (NC) shRNA were used to transfect SW10 cells. The knockdown efficiency of the shRNA was detected using Western blotting.

### Conditioned medium collection and ELISA assay

SW10 cells (5 × 10^5^) were seeded into the 60 mm culture dish with different treatments. Then, the cells were washed with serum-free medium (SFM) and cultured for another 24 h with SFM (5 ml). Afterward, the media were centrifuged to collect the supernatants. The prepared CM was used immediately or stored at −80 °C for the following experiments. For quantitative analysis of AM in media, the RayBio Human AM ELISA Kit (Norcross, GA, USA) was used following the manufacturer’s protocol.

### *In vitro* mast cell recruitment assay

The 24-well transwell assay was used to detect mast cell recruitment with 1 × 10^5^ mast cells in the upper chamber and CM or SW10 cells in the lower chamber. SW10 cells in the lower chamber was previously treated with indicated treatment for 24 h, washed with SFM and counted to make sure the same number of cells added into the lower chamber. The fibronectin (10 μg/ml, sc-29011 Santa Cruz Biotechnology) covered upper chamber. We incubated the chambers (37 °C, 4 h) before scraping and washing the filters with PBS. Then, cells on the filters were fixed (4% paraformaldehyde), stained (0.1% crystal violet) and counted to evaluate mast cell recruitment. The results are showed as mean ± SD.

### *In vivo* mast cell recruitment assay

shNf1-SW10 cells (3 × 10^6^) were suspended in medium containing Matrigel (1:4; BD Biosciences, San Jose, CA, USA) and subcutaneously injected into the right dorsal of BALB/c nude mice (4-week-old, male). One week later, xenografts reached a volume of 0.5 cm^3^ and the mice were randomly divided into three groups (n = 6). Mice were intraperitoneally injected with DHT (0.6 mg/kg), MDV3100 (1 mg/kg), or intratumourally injected with dimethylsulphoxide (10%). Red fluorescent protein (RFP)-labelled HMC-1 cells (5 × 10^6^) were injected via the tail vein of the tumour-bearing mice 48 h previous to tumour harvest. The tumours were prepared in frozen sections and paraffin-embedded sections for subsequent analysis with fluorescence microscopy and immunohistochemistry (IHC) staining with anti-AM and YAP. All protocols for animal experiments were approved by the institutional review board of the First Affiliated Hospital of Xi’an Jiaotong University.

### RNA extraction and real-time quantitative PCR (qRT-PCR) assay

The Total RNA Extraction Kit (Fastagen Biotech, Shanghai, China) was used to extract total RNA from cells. The extracted RNA (1 μg) was used as a template for reverse transcription with Superscript III transcriptase (Invitrogen, Eugene, OR, USA). For the analysis of gene expression at the mRNA level, qRT-PCR was performed by using the Bio-Rad CFX96 system with SYBR Green. The mRNA level of glyceraldehyde-3-phosphate dehydrogenase (GAPDH) was employed as the internal control. Primer sequences for the gene of interest can be found in Supplementary Table S1.

### Extraction of nuclear and cytoplasmic proteins

The NE-PER Nuclear Cytoplasmic Extraction Reagent kit (Pierce, Rockford, IL, USA) was used to isolate and extract nuclear and cytoplasmic proteins. Briefly, cells were washed with PBS before centrifugation (500 g, 3 min). Then, the supernatants were discarded, and the precipitates were suspended in cytoplasmic extraction reagent I (200 μl) and vortexed. The suspensions were incubated for 10 min on ice prior to the addition of cytoplasmic extraction reagent II (11 μl). The suspensions were vortexed for 5 s, followed by incubation for 1 min on ice and centrifugation (16 000 g, 5 min). The supernatants were transferred into cold tubes (cytoplasmic extracts). The precipitates were suspended using nuclear extraction reagent (100 μl) and vortexing, followed by incubation (on ice, 10 min). Then, the mixtures were centrifuged for 10 min (16 000 g), and the supernatants were collected (nuclear extracts).

### Western blot assay

RIPA lysis buffer including protease inhibitors was used to lyse cells before centrifugation (15 000 g, 15 min). The supernatant was collected (total protein extract), and the concentration of the extracted protein was analysed using the Bradford method. Briefly, 30 μg of protein was electrically separated in SDS-polyacrylamide gel (12%) and transferred onto nitrocellulosefilter membranes. Skim milk (5%) incubated the membranes at room temperature for 1 h to block non-specific binding sites. Then, primary antibodies (16 h, 4 °C) and secondary antibodies (1 h, room temperature) incubated the membranes separately. A Molecular Imager (Bio-Rad Laboratories Inc., Hercules, CA, USA) was used to visualize the protein bands. Western blotting of GAPDH was employed as the internal control.

### Clinical specimens and IHC

To study mast cells in cutaneous neurofibroma (cNF) tissues, 40 dermal neurofibroma tissues and adjacent cutaneous tissues were collected from patients who underwent tumour resection from June 2010 to October 2017 at the First Affiliated Hospital of Xi’an Jiaotong University. Before the sample collection, approval from the institutional review board was obtained from the First Affiliated Hospital of Xi’an Jiaotong University; collection was performed with informed consent from every patient or from a parent of patients under 18. The experiment was performed in accordance with the 1964 Declaration of Helsinki and its later amendments or comparable ethical standards. Tissues were fixed (4% paraformaldehyde), embedded with paraffin, and cut into sections (five micrometer) for IHC staining with a DAKO Autostainer Plus system. The sections were deparaffinized, rehydrated and heated to retrieve the antigen. Methanol containing 3% H_2_O_2_ blocked peroxidase and alkaline phosphatase activity. Primary antibodies against tryptase (dilution 1:200), AR (dilution 1:200), AM (dilution 1:150) and YAP (dilution 1:200) incubated sections overnight at 4 °C. Then, the sections were washed with PBS and incubated with secondary antibodies (at room temperature, 30 min). Afterward, diaminobenzidine and haematoxylin were incubated with the sections. One pathologist examined every section in a double-blinded manner under a high-power field (×400). The average number of tryptase-positive cells in 5 random fields was defined as mast cell density.

### Chromatin immunoprecipitation assay

The SimpleChIP Enzymatic Chromatin IP Kit (magnetic beads), purchased from Cell Signaling Technology, was used for the ChIP assay. Cells were prepared for the ChIP assay according to the manufacturer’s protocol. The protein/DNA complex was precipitated with antibodies against IgG (negative control), histone 3 (positive control), or YAP/TEAD. Region-specific primers were prepared to determine the precipitated DNA using PCR. All the region-specific primers are shown in Supplementary Table S2.

### Oligonucleotides pull-down assay

Primers labeled with biotin (GENEWIZ, China, Supplementary Table S3) on the 5′-end were synthesized to amplify DNA fragment of the binding site on AM promoter (−541 to −300). Cells were lysed, and cell extracts were collected. ImmunoPure streptavidin-agarose beads (20 μl/sample, Pierce) were incubated with the cell extracts at 4 °C for 1 h before centrifugation (1 min, 5000 g). Biotinylated oligonucleotides (100 pmol) and poly(dI-dC)·poly(dI-dC) (10 μg) were added to the collected supernatant for another 24 h incubation at 4 °C. Afterward, immobilized streptavidin-agarose beads (30 μl) were added to the above mixture to obtain the DNA-bound proteins (4 °C, 1 h). Then, the collected DNA-binding proteins were washed with lysis buffer prior to Western blotting analysis.

### Dual luciferase activity assay

pGL3-basic plasmids containing a region of AM promoter (1100 bp) or a mutant oligonucleotide of above region were used as the AM promoter report plasmids (pGL3-AM) and control report plasmids (pGL3-control). X-tremeGENE HP DNA transfection reagent (Roche, Mannheim, Germany) was used to transfect plasmids together with YAP into cells. Cells were treated with MDV3100, DHT or DMSO for 24 h before the luciferase assay. The Dual Luciferase Assay Kit (Promega, Madison, WI, USA) was used for the luciferase assay according to the manufacturer’s protocol. The experiments were repeated three times.

### Statistical analysis

For statistical analysis between two groups, Student’s t-test was employed using GraphPad Prism version 5.0 software (San Diego, CA, USA). For comparison of three groups, one-way ANOVA and LSD t-test (Fisher’s least significant difference t-test) were employed using SPSS (for Windows 10.0). For the correlation analysis, Spearman’s correlation test was used with SPSS. A *P* < 0.05 was defined as statistically significant.

## Supplementary information

supplementary information

## Data Availability

No datasets were analysed or generated in the present study.
