# Supplementary material for: AR facilitates YAP-TEAD interaction with the AM promoter to enhance mast cell infiltration into cutaneous neurofibroma
Source: Sci Rep. 2019 Dec 18;9:19346. doi: 10.1038/s41598-019-56022-9 (PMC6920444; doi:10.1038/s41598-019-56022-9)
Supplement: Supplementary file 1 — supplementary information [file 41598_2019_56022_MOESM1_ESM.doc]

**AR facilitates YAP-TEAD interaction with the AM promoter to enhance mast cell infiltration into cutaneous neurofibroma**

Jing Jia^1,2^, Haibao Zhang^3^, Hongke Zhang^1^, Wenbo Liu^1^, Huicong Du^1^, Maoguo Shu^1,*^, Lin He^1,*^

^1^Department of Plastic, Cosmetic and Maxillofacial, The First Affiliated Hospital of Xi’an Jiaotong University, Xi’an, Shaanxi, China

^2^The School of Electronic and Information Engineering, Xi’an Jiaotong University, Xi’an, Shaanxi, China

^3^Key Laboratory for Tumor Precision Medicine of Shaanxi Province, Xi’an, Shaanxi, China

**Supplementary Table 1. Primer sequence**

| AM | forward | TTGCCAGTGGGACGTCTGAG |
| --- | --- | --- |
|  | reverse | GTACATCAGGGCGACGGAAAC |
| CCL2 | forward | CTTCTGTGCCTGCTGCTCATA |
|  | reverse | CTTTGGGACACTTGCTGCTG |
| CCL5 | forward | ACCAGTGGCAAGTGCTCCAAC |
|  | reverse | TCTCCATCCTAGCTCATCTCCAAAG |
| VEGF | forward | GAGCCTTGCCTTGCTGCTCTA |
|  | reverse | CACCAGGGTCTCGATTGGATG |
| PD-ECGF | forward | GAGGCACCTTGGATAAGCTGGA |
|  | reverse | CTGCTCACTCTGACCCACGATAC |
| FGF | forward | GTGTGCTAACCGTTACCTGGCTATG |
|  | reverse | CCAGTTCGTTTCAGTGCCACA |
| SCF | forward | CCAGTTCGTTTCAGTGCCACA |
|  | reverse | GCAGCTGAAGATAAATGCAAGTGAG |
| GAPDH | forward | ATGGGGAAGGTGAAGGTCGG |
|  | reverse | GACGGTGCCATGGAATTTGC |

**Supplementary table2. Sequence of primer for ChIP assay**

| AM set | forward | ATACTTTGGTCCGGGGTTGG |
| --- | --- | --- |
|  | reverse | ATTCCGCTCTCCTCTGACGC |

**Supplementary** Table 3. Primer sequence of Oligonucleotides pull-down assay.

| AM set | forward | 5’-Biotin- ATACTTTGGTCCGGGGTTGG -3’ |
| --- | --- | --- |
|  | reverse | 5’-Biotin- ATTCCGCTCTCCTCTGACGC -3’ |

**Supplementary Figure 1:**

**
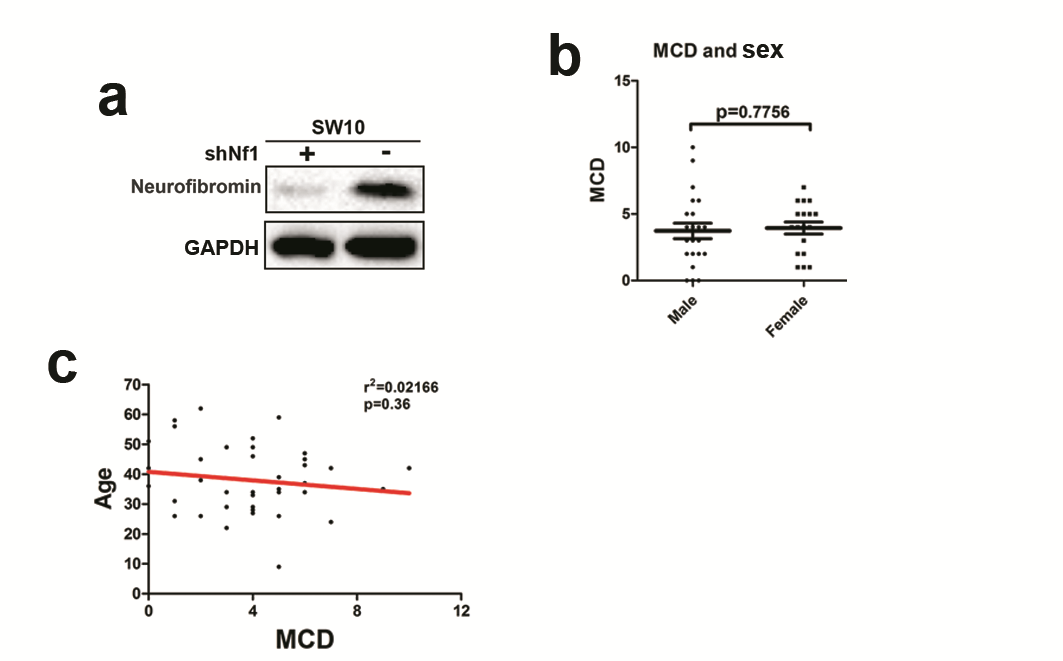
**

**Supplementary Fig. 1. Nf1 was successfully knocked down in shNf1-SW10 cells, and MCD had no correlation with sex or age.** (**a**) Western blot analysis showed low neurofibromin levels in shNf1-SW10 cells. (**b**) Comparison of MCD in neurofibroma tissues of different sexes. (**c**) Correlation analysis of age and MCD by linear regression.

**Supplementary Figure 2:**

**
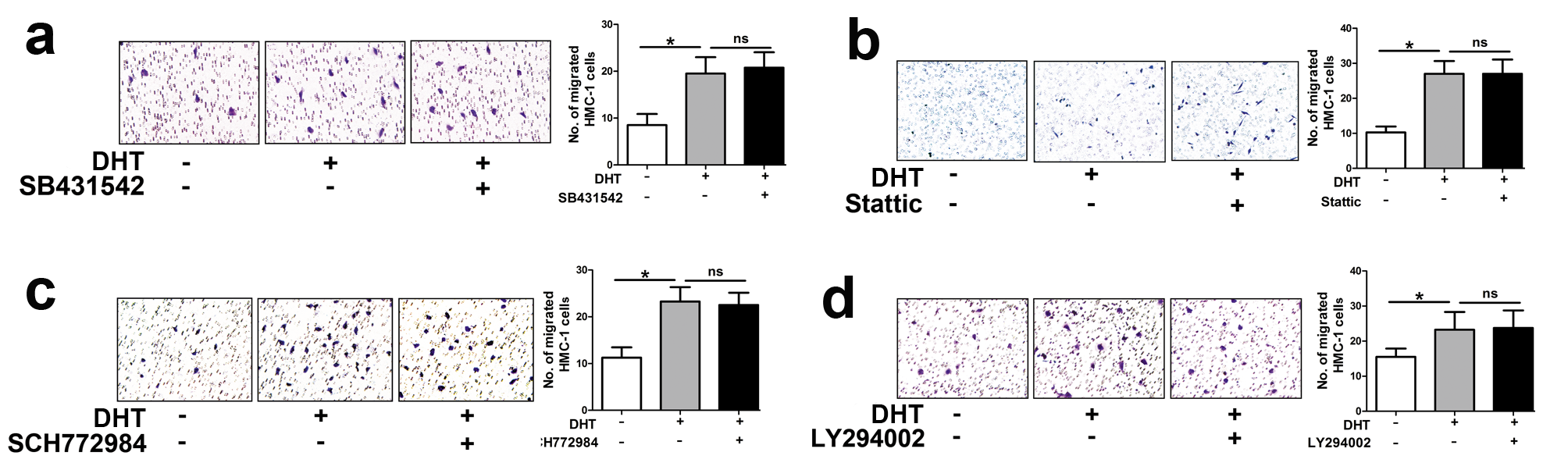
**

**Supplementary Fig. 2. Inhibition of the TGF-β, JNK/Stat3, MAPK or PI3K/Akt pathways failed to suppress DHT-induced HMC-1 recruitment.** (**a**) No significant alteration of HMC-1 recruitment was found in DHT-treated SW10 cells with or without SB431542 (10 μM) treatment. (**b**) DHT-treated SW10 cells enhanced HMC-1 infiltration, while Stattic (inhibitor of JNK/Stat3 signalling, 5 μM) treatment did not alter that enhancement. (**c**) Inhibition of the MAPK pathway (SCH7712984, 10 μM) failed to reverse DHT-induced HMC-1 migration. (**d**) PI3K/Akt pathway inhibitor (LY294002, 10 μM) failed to impair HMC-1 migration induced by DHT. **P*<0.05.

**Supplementary Figure 3:**


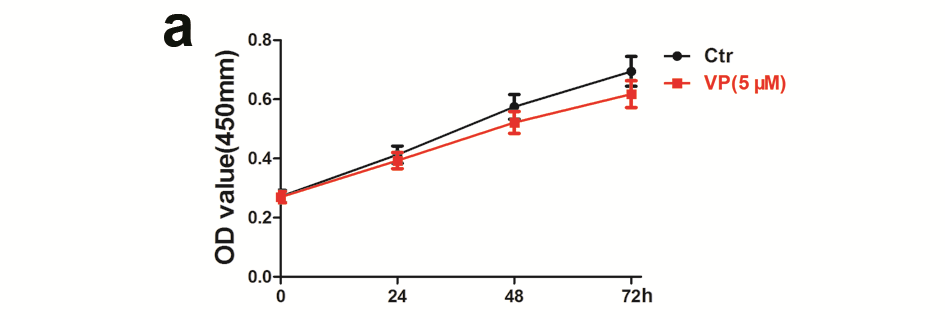


**Supplementary Fig. 3. VP only slightly repressed cell viability in shNf1-SW10 cells.** (**a**) Cell viability of shNf1-SW10 cells treated with VP (5 μM) at different time points. **P*<0.05.

**Supplementary Figure 4:**


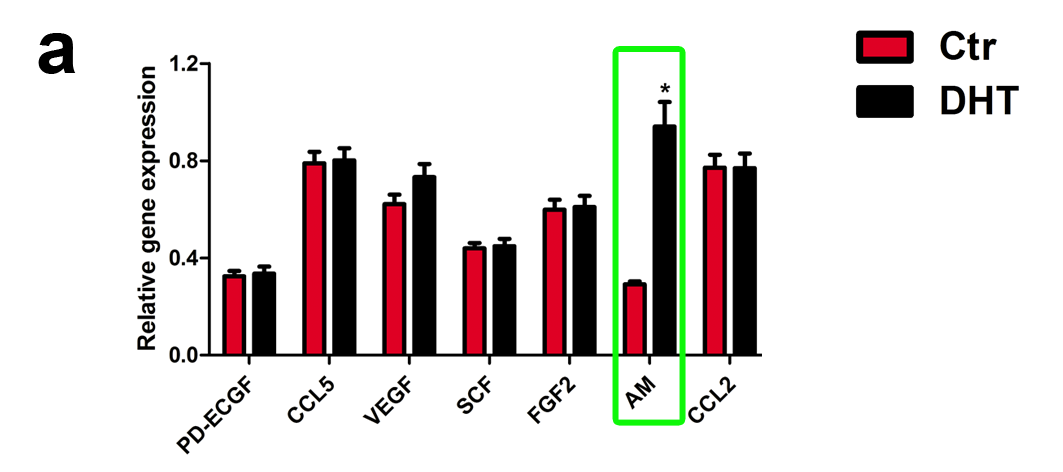


**Supplementary Fig. 4. DHT upregulated AM expression in SW10 cells.** (**a**) SW10 cells were treated with DHT for 24 h before detecting the mRNA levels of the indicated genes. **P*<0.05.

**Supplementary Figure 5：**

**
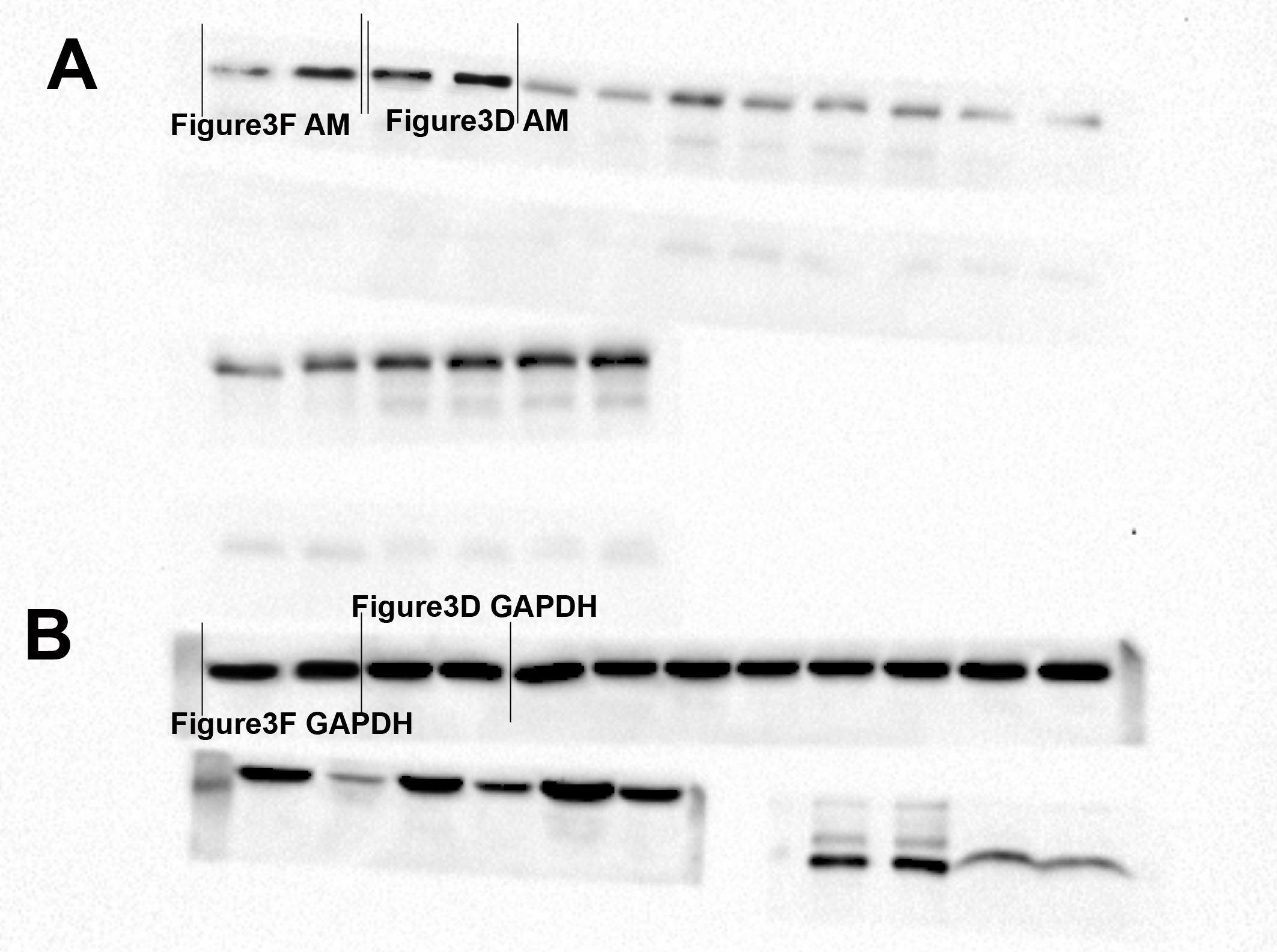
**

**Supplementary Figure 5: Full Western Blots for Figure 3.** (A) Full-length exposures of AM in DHT-treating SW10 cells. (B) Full-length exposure of AM in AM-overexpressing SW10 cells.

**Supplementary Figure 6:**

**
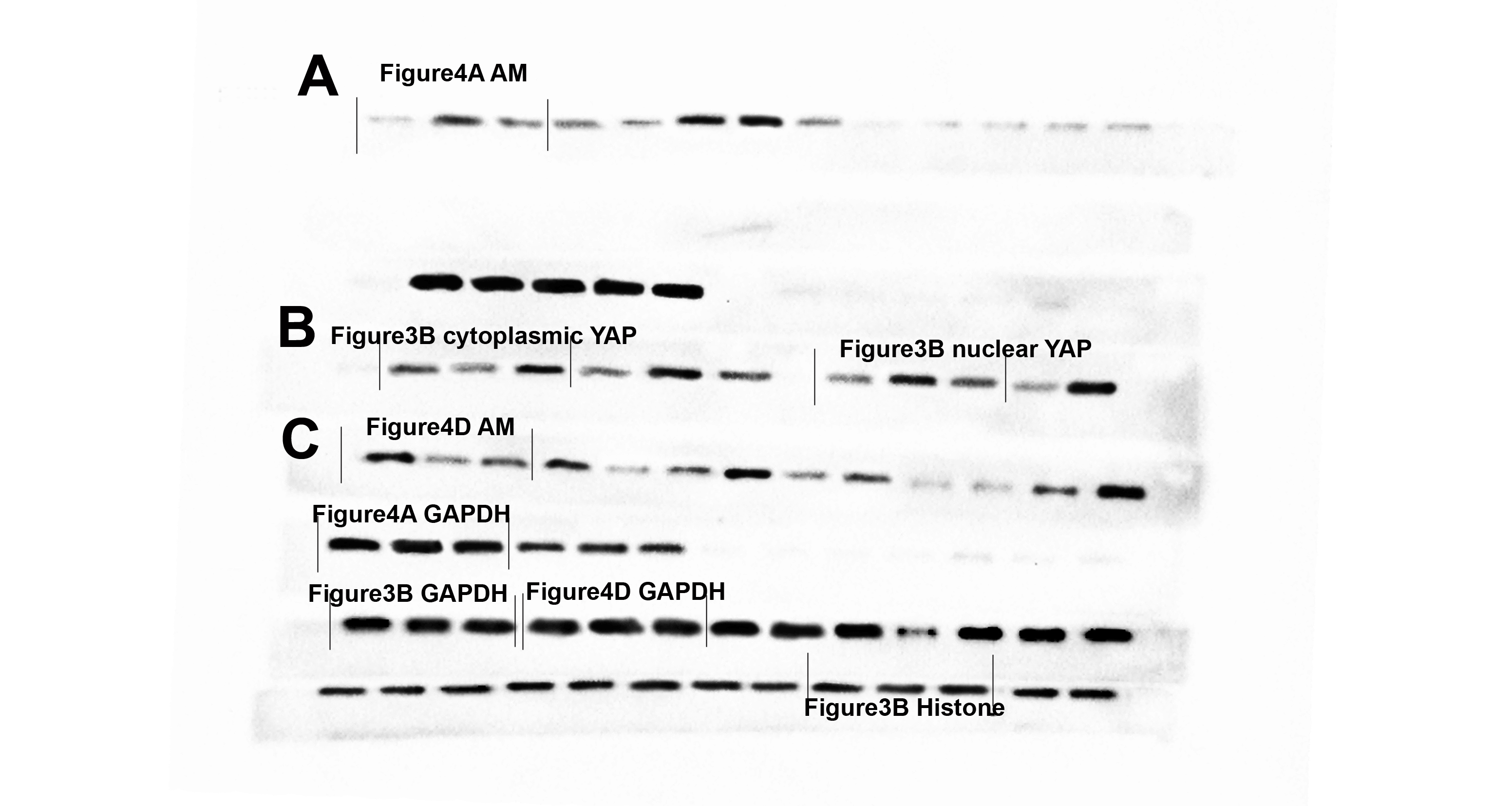
**

**
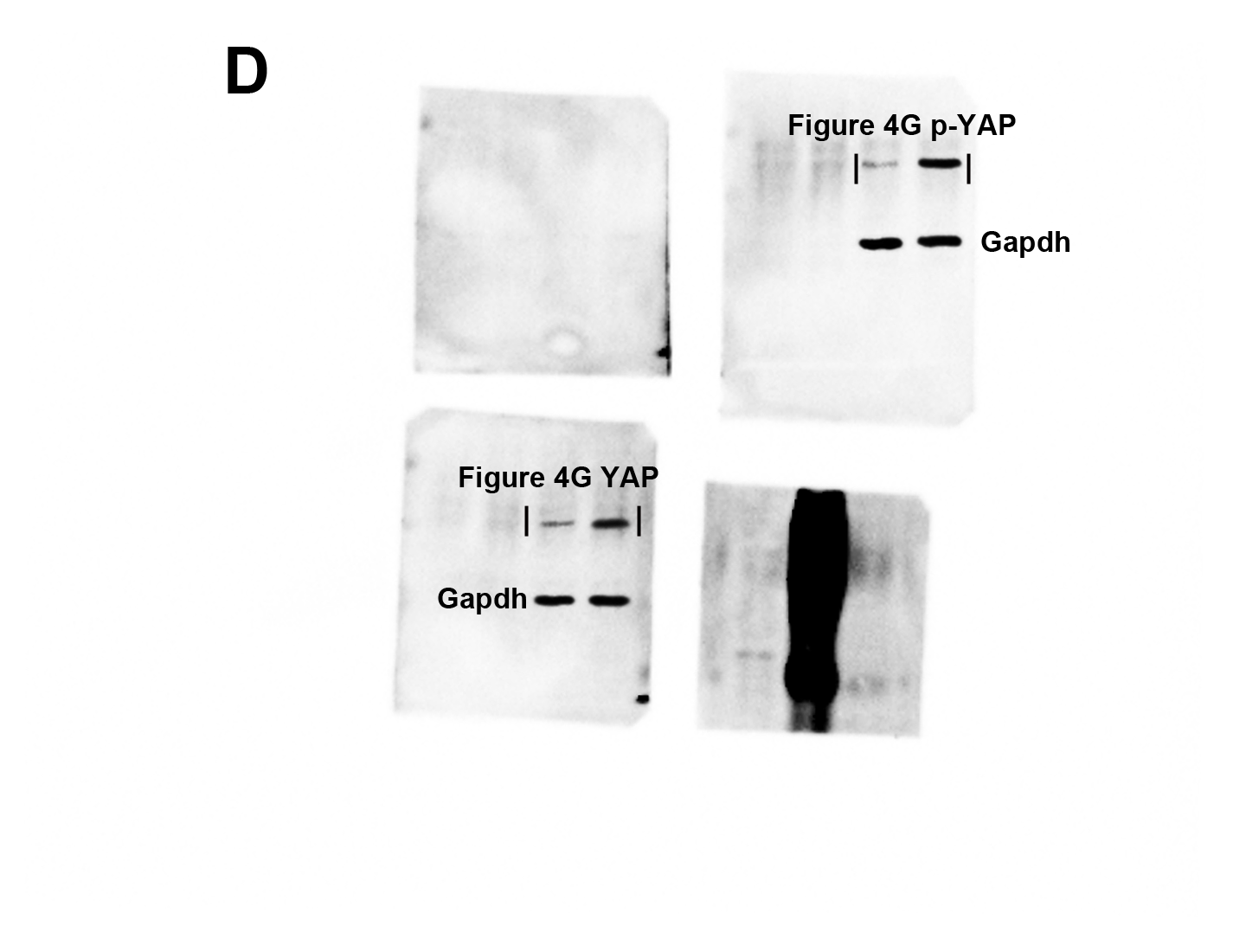
**

**
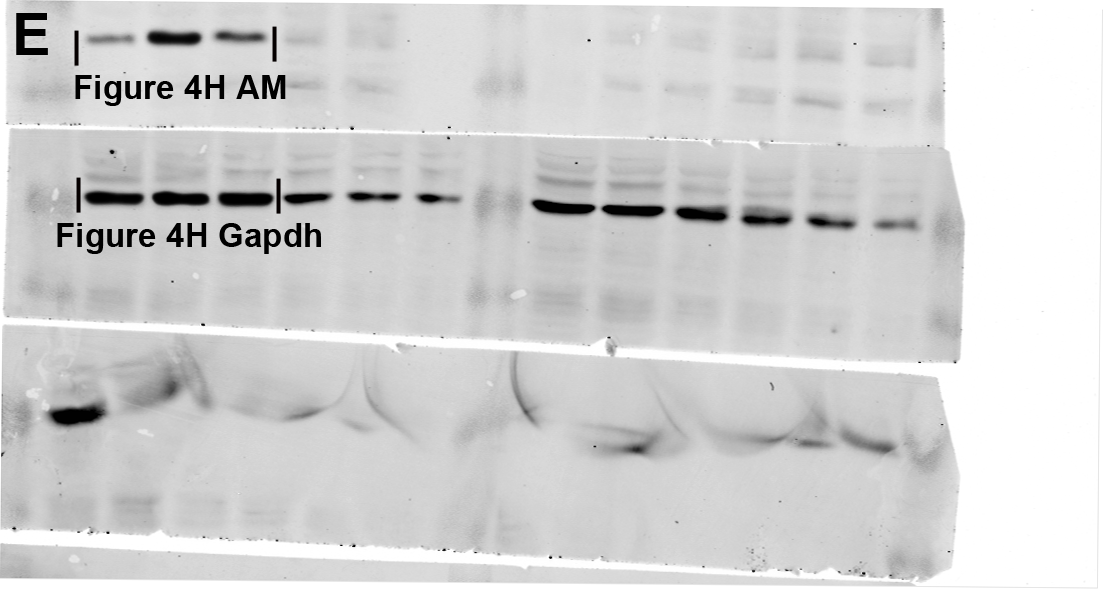
**

**Supplementary Figure 6: Full Western Blot for Figure 3 and Figure 4.** (A) Protein level of AM with treatment of DHT separately or with VP corporately. (B) Protein level of nuclear and cytoplasmic YAP in SW10 cells treated with DHT or MDV3100. (C) Protein level of AM in DHT-treating SW10 cells. (D) Protein level of p-YAP and YAP in shNf1-SW10 cells with or without YAP knockdown. (E) Protein level of AM with treatment of DHT separately or with YAP knockdown corporately.

**Supplementary Figure 7:**


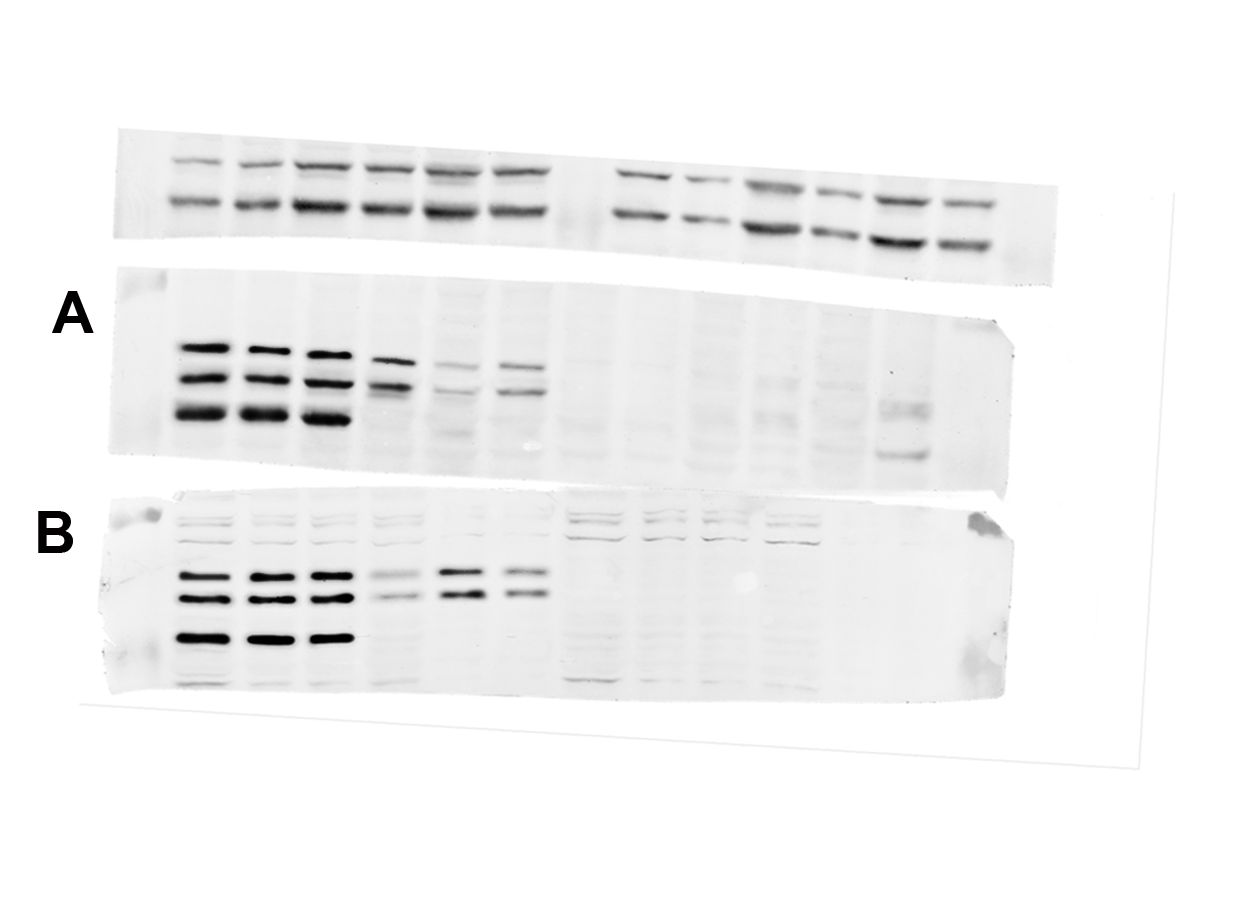


**Supplementary Figure 7: Full Western Blot for Figure 3 and Figure 5.** (A) Full western of oligonucleotides pull-down assay in cells treated with MDV3100 with or without XMU-MP-1. (B) Full western of oligonucleotides pull-down assay in cells treated with DHT with or without VP.

**Supplementary Figure 8:**

**
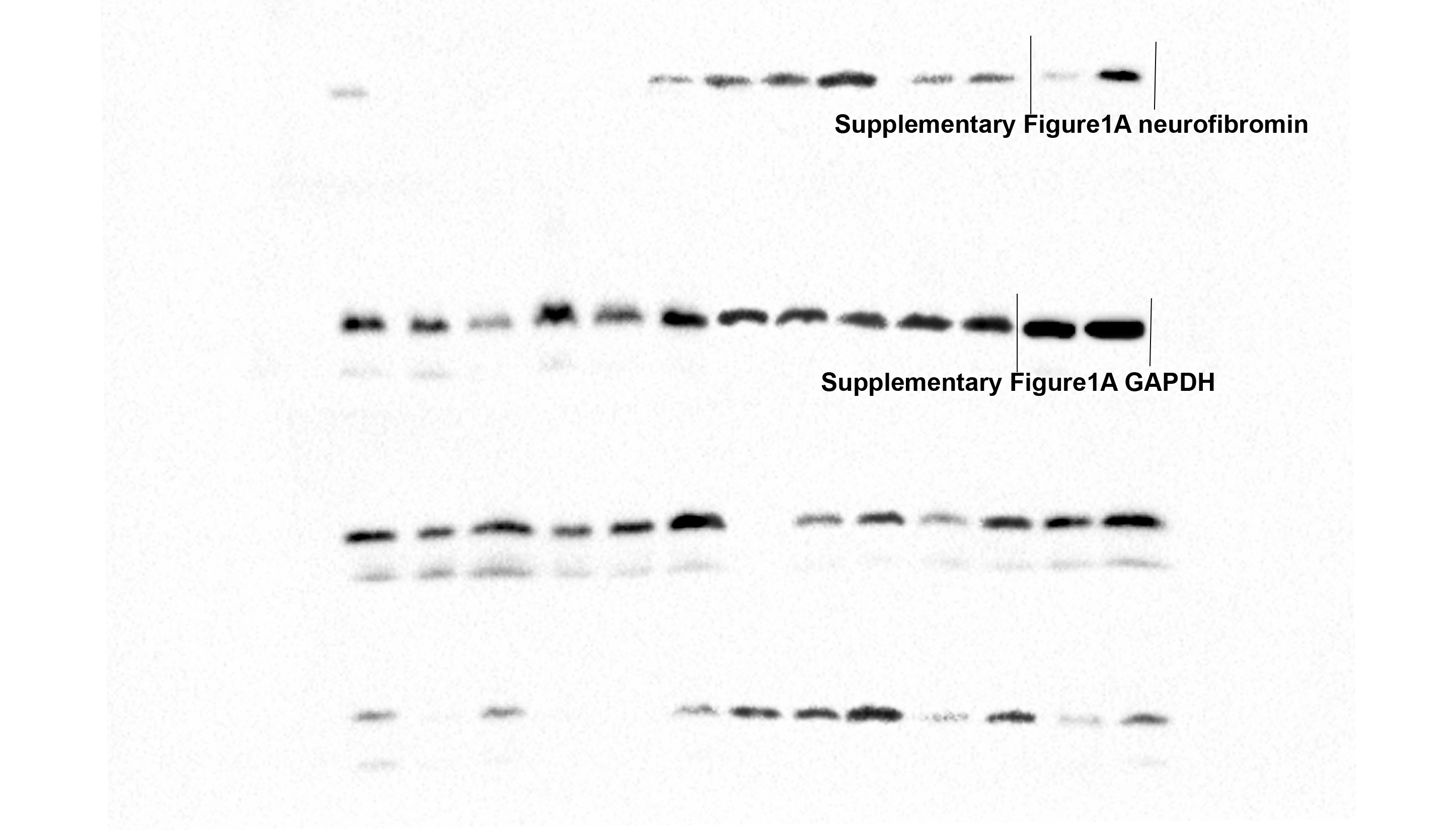
**

**Supplementary Figure 8: Full Western Blot for Supplementary Figure 1.** Protein level of neurofibromin in SW10 cells with or without Nf1 knockdown.
